# Molecular epidemiological analyses reveal extensive connectivity between *Echinostoma revolutum* (*sensu stricto*) populations across Eurasia and species richness of zoonotic echinostomatids in England

**DOI:** 10.1371/journal.pone.0270672

**Published:** 2023-02-06

**Authors:** Egie E. Enabulele, Scott P. Lawton, Anthony J. Walker, Ruth S. Kirk

**Affiliations:** 1 Molecular Parasitology Laboratory, School of Life Sciences, Pharmacy and Chemistry, Kingston University, Kingston upon Thames, Surrey, United Kingdom; 2 Epidemiology Research Unit, Department of Veterinary and Animal Sciences, Northern Faculty, Scotland’s Rural College, Inverness, United Kingdom; Universidade Federal de Minas Gerais, BRAZIL

## Abstract

*Echinostoma revolutum* (*sensu stricto*) is a widely distributed member of the Echinostomatidae, a cosmopolitan family of digenetic trematodes with complex life cycles involving a wide range of definitive hosts, particularly aquatic birds. Integrative taxonomic studies, notably those utilising *nad*1 barcoding, have been essential in discrimination of *E*. *revolutum* (*s*.*s*.) within the ‘*Echinostoma revolutum*’ species complex and investigation of its molecular diversity. No studies, however, have focussed on factors affecting population genetic structure and connectivity of *E*. *revolutum* (*s*.*s*.) in Eurasia. Here, we used morphology combined with *nad*1 and *cox*1 barcoding to determine the occurrence of *E*. *revolutum* (*s*.*s*.) and its lymnaeid hosts in England for the first time, in addition to other echinostomatid species *Echinoparyphium aconiatum*, *Echinoparyphium recurvatum* and *Hypoderaeum conoideum*. Analysis of genetic diversity in *E*. *revolutum* (*s*.*s*.) populations across Eurasia demonstrated haplotype sharing and gene flow, probably facilitated by migratory bird hosts. Neutrality and mismatch distribution analyses support possible recent demographic expansion of the Asian population of *E*. *revolutum* (*s*.*s*.) (*nad*1 sequences from Bangladesh and Thailand) and stability in European (*nad*1 sequences from this study, Iceland and continental Europe) and Eurasian (combined data sets from Europe and Asia) populations with evidence of sub-population structure and selection processes. This study provides new molecular evidence for a panmictic population of *E*. *revolutum* (*s*.*s*.) in Eurasia and phylogeographically expands the *nad*1 database for identification of echinostomatids.

## Introduction

The Echinostomatidae is a diverse, widely distributed family of hermaphrodite digenetic trematodes comprising 37 nominal genera, typically characterized by a circumoral collar of spines and a spiny tegument [1]. The adults commonly infect the intestine and bile ducts of aquatic or semi-aquatic birds and mammals [2, 3]. Some echinostomatid species cause disease in humans in specific foci in East and Southeast Asia as a result of ingestion of metacercariae in raw or undercooked molluscs, fish, crustaceans and amphibians [3]. Reports of such cases outside of Asia are limited, although accurate epidemiological mapping is undermined by misidentifications and diagnostic challenges [2, 3]. Furthermore, certain echinostomatid species are important pathogens of animals and have been associated with mortalities or severe pathology in amphibians [4, 5]. Host-parasite relationships of the Echinostomatidae have been extensively investigated as experimental models [6] and in ecological studies [7], particularly since echinostomes can castrate, reduce longevity and influence growth (stunting or gigantism) of first intermediate snail hosts [8].

There is a long history of controversy about the systematics of the Echinostomatidae due to inter-specific homogeneity of morphological characteristics, loss/unavailability of type material and incorrect identification of sequenced isolates resulting in extensive synonymies, nomenclatural issues and a requirement for systematic revisions [9]. Taxonomy of the cryptic 37-collar-spined ‘*Echinostoma revolutum*’ group has been particularly difficult to resolve [reviewed in 10]. Integrative taxonomic studies utilising morphological and molecular methods have discriminated genetically distinct echinostomatids worldwide [11–21]. Many studies have used the mitochondrial DNA barcoding marker *NADH dehydrogenase subunit* 1 (*nad*1) gene as it is the most informative locus for species discrimination of echinostomatids and inferences of phylogenetic relationships [14, 15, 22]. Numerous species were previously synonymised with *E*. *revolutum* [10]. The validity of *E*. *revolutum* and *E*. *miyagawai* as separate species was confirmed by morphometric [23], life cycle [24] and molecular differentiation [15, 17, 25]. Phylogenetic analyses of *nad*1 support the separation of *E*. *revolutum* into two cryptic species, Eurasian *E*. *revolutum* (*sensu stricto*) (as previously defined by Georgieva et al. [14] for *E*. *revolutum* from Europe) and North American *E*. *revolutum* [17, 18]. Isolates previously identified as *E*. *revolutum* (*E*. *friedi* from Valencia, Spain, Marcilla et al. Genbank; German isolate [22]) clustered with European [15] and Eurasian *nad*1 isolates of *E*. *miyagawai* [17, 21]. Australian isolates identified as *E*. *revolutum* [26] formed an Australasian lineage of *E*. *miyagawai* with an isolate [16] from New Zealand [21]. *Echinostoma friedi* was synonymised with *E*. *miyagawai* based on morphological [27] and molecular analysis [15]. Chai et al. [21] demonstrated that Bangladesh *E*. *robustum* [18] and Indonesian *E*. *miyagawai nad*1 isolates clustered with *E*. *miyagawai* sequences from Europe supporting synonymy of *E*. *miyagawai* with *E*. *robustum* and *E*. *friedi*. American sequences allocated to *E*. *robustum* probably belong to other species. For example, Brazilian *E*. *robustum* [12] is conspecific with *E*. *pseudorobustum* recently described in Brazil [20]. Finally, comparative mitogenomics have demonstrated the close genetic relationship of 37-collar-spined ‘*Echinostoma revolutum*’ members (*E*. *revolutum* (*s*.*s*.), *E*. *miyagawai*, *E*. *caproni* and *E*. *paraensei*), particularly the monophyletic position of *E*. *revolutum* (*s*.*s*.) to *E*. *miyagawai* [28].

*Echinostoma revolutum* (*s*.*s*.) is a widely distributed zoonotic echinostomatid [10]. Molecular tools have enabled its taxonomic relationships and genetic diversity to be investigated, but no studies have focused on the factors affecting population genetic structure and connectivity of the parasite in Eurasia. We aimed to address this gap in knowledge in the present study. Initially, we sought to accurately identify the occurrence of *E*. *revolutum* (*s*.*s*.) and other echinostomatid species and their intermediate snail hosts in the UK by *nad*1 and *cox*1 barcoding, respectively. Secondly, using phylogeographical approaches and currently available data, we aimed to determine the relationship of the UK population of *E*. *revolutum* with populations from Europe and Southeast Asia. This enabled connectivity between parasite populations to be evaluated and provided insights into parasite movement and population structure. Finally, we used the *nad*1 data to provide an initial assessment of demographic changes in the parasite population and to identify signatures of selection which may account for the patterns seen in population structuring. Collectively, this study provides a unique insight into the molecular epidemiology of *E*. *revolutum* (*s*.*s*.) and reveals panmixia across Eurasia, probably facilitated by migratory bird hosts dispersing the parasites between susceptible snail populations.

## Materials and methods

### Sample collection

A total of 995 snails (Lymnaeidae, Physidae, Planorbidae) were collected by hand netting from the littoral zone of 15 freshwater sites in England and five in Scotland from July 2013-August 2014 as part of an ongoing study on diversity of digeneans and their intermediate snail hosts [29–31]. Permission to collect snails was granted by landowners of all sites. They were initially identified using shell morphology [32]. In the laboratory, snails were individually placed in dechlorinated tap water filtered through a carbon filter (Silverline, UK) in 6-well plates (snails < 30 mm) or in 50 ml beakers (snails > 30 mm), exposed to natural light and checked for emerging cercariae. Non-shedding snails were incubated in the dark for up to 72 h and re-exposed to light. Snails were then dissected and checked for prepatent (larval) infections. Only lymnaeid snails from nine English sites were infected with echinostomatids (Table 1) and their identification was further evaluated using *cox*1 DNA barcoding and phylogenetic approaches employed by us previously [33, 34] (S1 and S2 Tables). Representative samples of each cercarial isolate (a group of identical individuals collected from a single host at one point in time [26]) were examined live with neutral red stain under a stereomicroscope for initial identification using a key to larval digeneans [35] and ~20 of each isolate were fixed in 4% formalin for morphological analysis. A pool of ~30 cercariae was stored separately in molecular grade ethanol for DNA extraction.

**Table 1:** Summary data on snail species from echinostomatid infected freshwater sites in England.

Location	Coordinates	Parasite species	Snail species	Sample size	No. infected	Prevalence (%)
Bury Lake, Hertfordshire	51°38’0.1’’N, 00°28’57’’W		*Lymnaea stagnalis*	20	0	0
		*Echinostoma revolutum* (*s*.*s*.)	*Radix auricularia*	8	3	38
			*Stagnicola* sp.	1	0	0
Hill Pond Marsh Farm Fishery, Surrey	51°09’39’’N, 00°38’08’’W		*Lymnaea stagnalis*	6	0	0
		*Echinostoma revolutum* (*s*.*s*.)	*Radix auricularia*	5	1	20
		*Echinoparyphium recurvatum*			1	20
Peg Pond, Richmond Park, Surrey	51°26’00’’N, 00°16’37’’W	*Echinostoma revolutum* (*s*.*s*.)	*Ampullaceana balthica*	17	2	11.8
		*Echinoparyphium recurvatum*			5	29.4
			*Stagnicola* sp.	5	0	0
Pen Ponds, Richmond Park, Surrey	51°26’30’’N, 00°16’35’’W	*Echinostoma revolutum* (*s*.*s*.)	*Radix auricularia*	79	1	1.3
		*Echinoparyphium recurvatum*			4	5.1
Queen’s River, Bushy Park, Surrey	51°24’42”N, 00°20’27”W	*Echinoparyphium recurvatum*	*Ampullaceana balthica*	83	7	8.4
			*Lymnaea stagnalis*	3	0	0
Rams Paddock Pond, Pensthorpe Natural Park, Norfolk	52°49’02’’N, 00°53’46’’W	*Echinostoma revolutum* (*s*.*s*.)	*Ampullaceana balthica*	76	2	2.6
		*Echinoparyphium aconiatum*			3	3.9
		*Echinoparyphium recurvatum*			11	14.5
		*Hypoderaeum conoideum*			1	1.3
Sharmans Lake, Pensthorpe Natural Park, Norfolk	52°49’13”N, 00°53’33”E	*Echinoparyphium aconiatum*	*Lymnaea stagnalis*	51	6	11.8
Tundry Pond, Hampshire	51°16’00’’N, 00°53’27’’W	*Echinostoma revolutum* (*s*.*s*.)	*Ampullaceana balthica*	2	1	50
		*Echinostoma revolutum* (*s*.*s*.)	*Lymnaea stagnalis*	80	13	16.3
		*Echinoparyphium aconiatum*			10	12.5
		*Echinoparyphium aconiatum*	*Radix auricularia*	56	3	5.4
		*Echinoparyphium recurvatum*			2	3.6
		*Hypoderaeum conoideum*			1	1.8
Wildlife Pond, London Wetland Centre, London	51°28’39’’N, 00°14’89’’W	*Echinostoma revolutum* (*s*.*s*.)	*Ampullaceana balthica*	30	1	3.3
		*Echinoparyphium recurvatum*			1	3.3
			*Lymnaea stagnalis*	1	0	0
		*Echinostoma revolutum* (*s*.*s*)	*Stagnicola* sp.	16	2	12.5
Totals				539	81	

### Morphological analysis

Formalin fixed cercariae from each cercarial isolate were stained with acetocarmine stain. Morphological features were examined and measured in micrometres and photomicrographs captured on a Nikon Eclipse NiE microscope using NIS-Elements BR software. Voucher specimens of cercariae stained with acetocarmine and mounted in DPX were deposited at the Natural History Museum (NHM), London, UK (registration numbers: NHM UK 2018.11.14.2–4, NHM UK 2021.11.16.9–11).

### DNA extraction, amplification and sequence generation

Total genomic DNA was extracted from cercariae using the Qiagen DNeasy Blood and Tissue Kit (Qiagen Inc. UK) following the manufacturer’s protocol. Polymerase chain reaction (PCR) amplifications of partial fragments of the *nad*1 gene were performed using previously described primers [22]. The PCR was performed in a 25 μl reaction using 12.5 μl Thermo–Start^R^ PCR master mix (0.625 Units of Taq DNA polymerase, 1X reaction buffer, 0.2 mM of each dNTP and 1.5 mM MgCl_2_), 5 μl of eluted 50 ng DNA template and 3.75 μl of 0.2 μM of each forward and reverse primers. The thermocycling profile comprised an initial denaturing at 94°C for 3 min, 30 cycles of denaturation at 94°C for 30 s, annealing at 48°C for 30 s and extension at 72°C for 2 min, with a final elongation time of 10 min at 72°C; 5 μl of each PCR amplicon was visualized in 1% agarose gels stained with gel red (Meridian Bioscience, USA). The remaining PCR products were sequenced with the same primers at the NHM, London, using fluorescent dye terminator sequencing kits (Applied Biosystems), then run on an Applied Biosystems 3730XL automated sequencer. Sequences were assembled and manually corrected for ambiguous base calls using BioEdit [36] and submitted to GenBank (S1 Table).

### Phylogenetic analyses

An initial BLAST(n) search on GenBank indicated that the 81 novel *nad*1 echinostomatid sequences were similar to *Echinostoma* (n = 26), *Echinoparyphium* (n = 53), and *Hypoderaeum* (n = 2). Novel and published *nad*1 sequences available in GenBank for *Artyfechinostomum malayanum* (syn. *Echinostoma malayanum*), *Echinoparyphium* spp., *Echinostoma* spp., *Hypoderaeum conoideum* and *Isthmiophora* spp. were aligned using MUSCLE [37] with *Fasciola hepatica* selected as an outgroup (S1 and S3 Tables). Phylogenetic analysis was inferred by Maximum Likelihood (ML) algorithms implemented in MEGA X [38] using 1,000 bootstrap replicates. Prior to ML analysis, MEGA X was used to determine the appropriate evolutionary models and the Hasegawa-Kishino-Yano model with estimates of invariant sites and gamma distributed among-site rate variation (HKY + I + G) was selected.

The *K/θ* species criterion was used to provide a quantitative assessment of the differentiation across the *nad*1 alignment between *E*. *revolutum* from the USA [12, 13] and England (this study) + Iceland + continental Europe [11, 14, 15] + Asia (Bangladesh and Thailand) [17, 18]. The *K/θ* species criterion was developed to differentiate between lineages of asexual organisms that do not undergo high frequency of genetic recombination. It was subsequently applied to distinguish between species that undergo sexual reproduction, using mitochondrial markers owing to their lack of recombination and high mutation rates [39, 40]. The *K/θ* model is designed to identify the ratio of divergence between individuals from separate clades/lineages separated by *t ≥4N*_*e*_ generations (where *t* is the time to most recent common ancestor and *N*_*e*_ is the effective population size), which is equivalent to the upper 95% confidence limit of the coalescent time and the depth of separation formed by random drift. For two such clades/lineages the ratio of divergence between individuals from each clade (*K*) and the Watterson estimator of population mutation rate (*θ*) is given by the equation *K*/*θ* = 2*tμ*/2*N*_*e*_*μ* ≥ 8*N*_*e*_*μ*/2*N*_*e*_*μ* = 4. Although we do not have an exact measure of *N*_*e*_ or the mutation rate (*μ*), these can be assumed to be similar for each clade/lineage and can be eliminated from the equation. Thus, the ratio of the mean pairwise sequence difference between a pair of clades (*K*) to the mean pairwise sequence difference within a clade (θ) can be used to determine whether the clades are samples from different species (K/θ≥4) or the same species (*K*/θ<4) with probability ≥0.95. The value of *θ is* derived from the mean sequence difference between individuals within a clade/lineage, *π*; this parameter can be calculated by *π*/(1 − 4*π*/3) [39, 40].

### Analyses of molecular diversity and population structure of *Echinostoma revolutum* (*s*.*s*.)

Novel and published sequence data were used to provide a broad indication of molecular diversity within and between populations of *E*. *revolutum* (*s*.*s*.) comprising representative *nad*1 sequences from countries in Europe and Asia (S4 Table). To assess connectivity and the occurrence of any underlying genetic population structure within Eurasian *E*. *revolutum* (*s*.*s*.), haplotype network analyses were performed using TCS implemented in PopART. A Mantel test was executed to identify any correlation between genetic distance and parasite populations. An analysis of isolate by distance was performed using ISOLDE implemented through Genepop (https://genepop.curtin.edu.au/genepop_op6.html) [41]. Pairwise genetic differentiation between sequences from different geographical locations was estimated using linearized F_st_ to construct a genetic distance matrix in DnaSP v6 [42]. Pairwise Euclidean distances between countries of origin were calculated measured in kilometres (km) using Google Earth to produce the geographical distance matrix.

To identify signatures of historical demographic alterations, Fu’s *Fs*, Tajima’s *D* and pairwise mismatch distribution analysis were calculated to identify the impact of non-neutral evolutionary forces on the Eurasian, European and Asian *E*. *revolutum* (*s*.*s*.) data sets using DnaSP v6. The distribution of the frequency of pairwise differences between sequences as a measure of mismatch distribution was performed for 1000 simulations, under the conditions of the constant growth model. The raggedness (*r*) statistic was used to measure the smoothness of the observed mismatch distribution to test for any significant departure from unimodality and to provide evidence of potential population expansion in the *E*. *revolutum* (*s*.*s*.) populations.

As sub-population structuring and natural selection can impact on the values of Fu’s *Fs*, Tajima’s *D* and *r*, the presence of selection in the *E*. *revolutum* (*s*.*s*.) *nad*1 was initially assessed by calculating the ratio of synonymous (dN) and non-synonymous (dS) substitutions across the entire alignment in DnaSPv6, where dN/dS <1 indicates purifying selection, dN/dS = 1 indicates neutrality and dN/dS >1 indicates the presence of positive selection. To account for the effect of selection between individual pairs of sequences, evidence of natural selection was calculated by estimating the average numbers of dN and dS between pairs of sequences under the Nei Gojobori method as implemented in MEGA X. The differences between dN and dS were calculated, and their significance tested by a two-tailed codon-based Z test to test the statistical hypothesis that dS = dN thus the observed polymorphisms would not be under selection (S5 Table). Provean (provean.jcvi.org/index.php) was used to investigate the impact of nucleotide substitutions on amino acid replacements in the *nad*1 protein and the likelihood of an amino acid substitution having a functional effect (default confidence threshold of -2.5) [43]. To identify where such mutations occurred, Protter (http://wlab.ethz.ch/protter/start/) [44] was employed to generate a 2D transmembrane model of the structure of the *nad*1 protein.

## Results

### Morphological identification of echinostomatid cercariae

**Fig 1:**
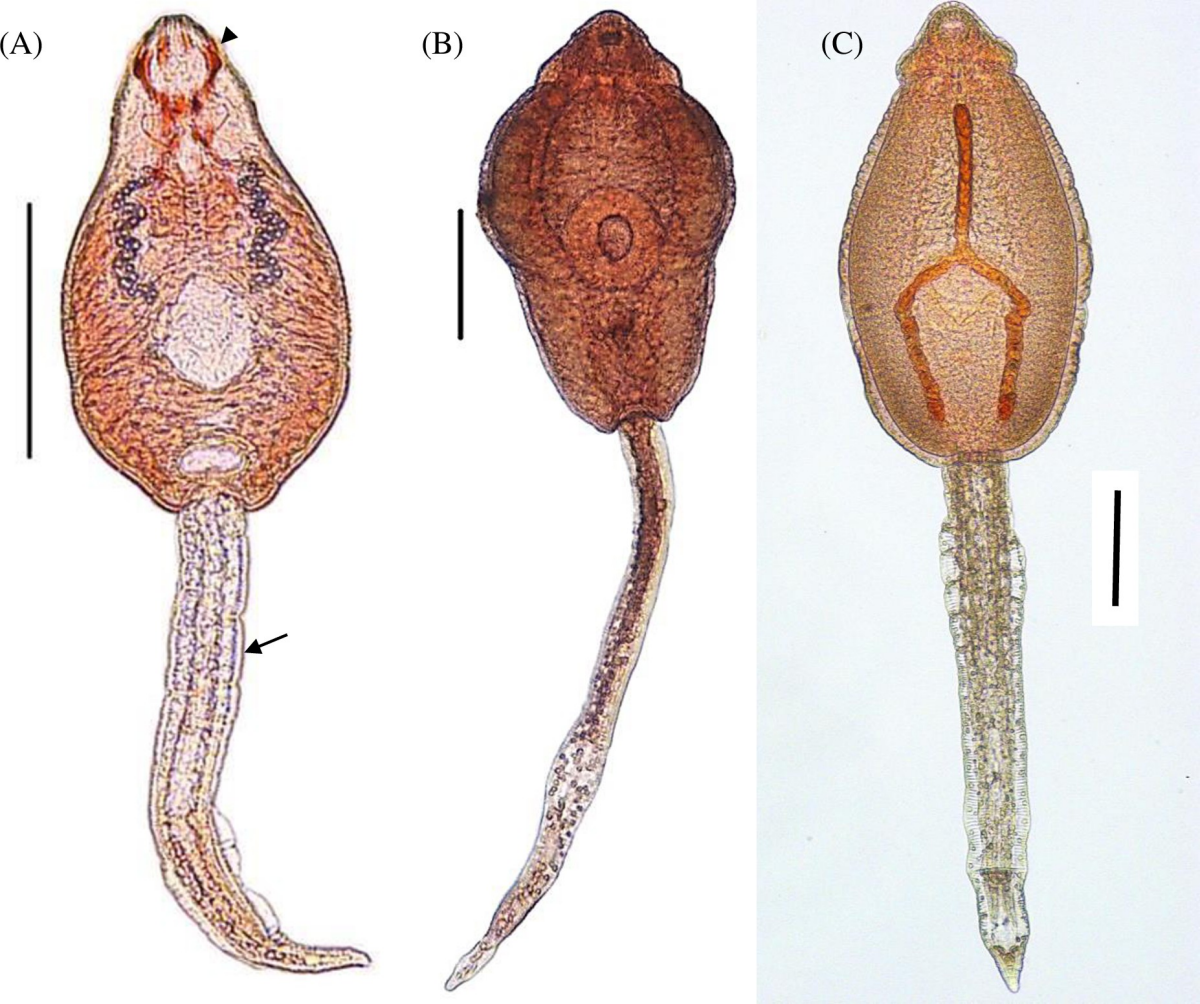
Photomicrographs of echinostomatid cercariae. (A) *Echinostoma revolutum* (*s*.*s*.) stained with neutral red to show outlets of paraoesophageal gland cells (arrowhead) and one of seven tegumental finfolds (arrow), scale-bar: 200 μm. (B) *Echinoparyphium recurvatum* and (C) *Echinoparyphium aconiatum* stained with acetocarmine, scale-bars: 100 μm.

Three species were morphologically identified: *E*. *revolutum* (*s*.*s*.), *Echinoparyphium aconiatum* and *Echinoparyphium recurvatum* (Fig 1, Table 1). Morphology of *E*. *revolutum* (*s*.*s*.) and *Ep*. *recurvatum* in this study corresponded with previous descriptions confirmed by *nad*1 sequences (*E*. *revolutum* (*s*.*s*.) [14, 27]; *Ep*. *recurvatum*: [19]), although morphometrics varied between studies (S6 Table). *Ep*. *aconiatum* morphology concurred with the key [35], but a detailed description associated with DNA sequences was not located in the published literature. Morphological identification agreed with molecular discrimination. *Hypoderaeum conoideum* was not morphologically examined as there were insufficient numbers of cercariae for examination due to low infections in two snail hosts.

### Phylogenetic identification of echinostomatid cercariae

**Fig 2:**
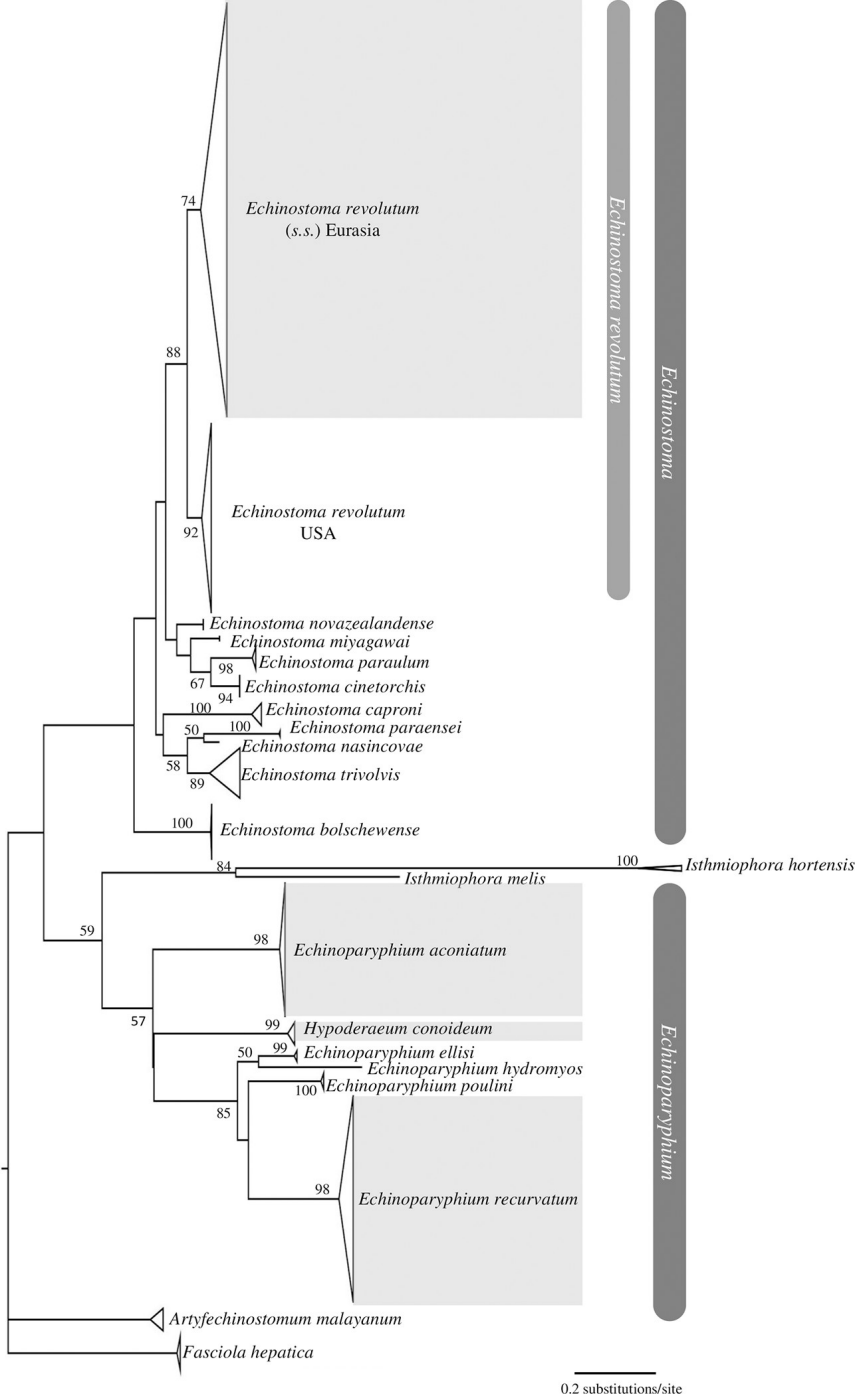
Phylogenetic tree based on maximum likelihood analysis of partial *nad*1 sequences for identification of echinostomatid cercariae from lymnaeid snails. Shaded clades indicate inclusion of species identified in this study. Species clades are collapsed. The scale-bar shows the number of nucleotide substitutions per site between DNA sequences. Nodal support values of ≥50 are shown.

In the present study, 81 partial *nad*1 novel sequences were generated (~480 base pairs each) for echinostomatid cercariae from lymnaeid snails (Table 1, S1 Table). BLAST searches on GenBank revealed four species with 99%-100% similarity to matched sequences: *E*. *revolutum* (n = 26); *Ep*. *aconiatum* (n = 22); *Ep*. *recurvatum* (n = 31) and *H*. *conoideum* (n = 2). The *nad*1 sequences generated here and retrieved from GenBank clustered in four strongly supported clades corresponding to *E*. *revolutum* (*s*.*s*.), *Ep*. *aconiatum*, *Ep*. *recurvatum* and *H*. *conoideum* in ML phylogenetic trees (Fig 2). *Hypoderaeum conoideum* phylogenetically nested within the *Echinoparyphium* clade. *Echinostoma revolutum* separated into two sub-clades, *E*. *revolutum* (*s*.*s*). Eurasia and *E*. *revolutum* of Detwiler et al. [2010] USA. *K*/θ≥4 confirmed Eurasian *E*. *revolutum* (*s*.*s*) as a genetically separate species from USA *E*. *revolutum* (Table 2). USA *E*. *revolutum* sequences were, therefore, excluded from molecular diversity analyses.

**Table 2:** Variables associated with *Echinostoma revolutum* (*sensu lato*) used to test compliance with the K/θ ratio for speciation.

Population	N	π	*θ*	*K*	*K/θ*
*Echinostoma revolutum* USA [12, 13]	35	0.01015	0.010289		5.9091
				0.061	
*Echinostoma revolutum* (*s*.*s*.) Eurasia (this study + Iceland + continental Europe [11, 14, 15] + Asia [17, 18])	73	0.01014	0.010290		5.9154

π = nucleotide diversity determined by the mean pairwise difference within *E*. *revolutum* sequences from the USA and *E*. *revolutum* from Eurasia (this study + Iceland + continental Europe + Asia) multiplied by the sample size correction n/(n– 1). N = number of sequences in each clade; θ = an estimator of which is equal to π /(1–4 /3); *K* = mean pairwise sequences difference between USA *E*. *revolutum* and Eurasia *E*. *revolutum*.

Echinostomatids infected 8.1% (81/995) of sampled snails. The 81 *cox*1 sequences clustered into four strongly supported clades for *Ampullaceana balthica* (junior synonyms *Lymnaea peregra*, *Radix balthica*, *Radix ovata*; https://www.molluscabase.org), *Radix auricularia*, *Lymnaea stagnalis* and a mixed cluster of *Stagnicola fuscus + Stagnicola palustris* (S1 Table, S1 Fig). The *cox*1 sequencing did not permit genetic differentiation of *Stagnicola* snails in this study, which are, therefore, referred to as *Stagnicola* sp. Molecular identification of snails was not always congruent with morphological identification due to phenotypic overlap of shell morphology between some specimens of *A*. *balthica* and *R*. *auricularia*. *Echinostoma revolutum* (*s*.*s*.) infected all four species of snails identified. *Echinoparyphium aconiatum* was hosted by three species: *A*. *balthica*, *R*. *auricularia*, *L*. *stagnalis*. *Echinoparyphium recurvatum* and *H*. *conoideum* were found in *A*. *balthica* and *R*. *auricularia*. Prevalence of infection in snails was variable and influenced by low sample sizes due to difficulty in locating snails in some freshwater sites (Table 1).

### Molecular diversity and population structure of *E*. *revolutum* (*s*.*s*.)

**Fig 3:**
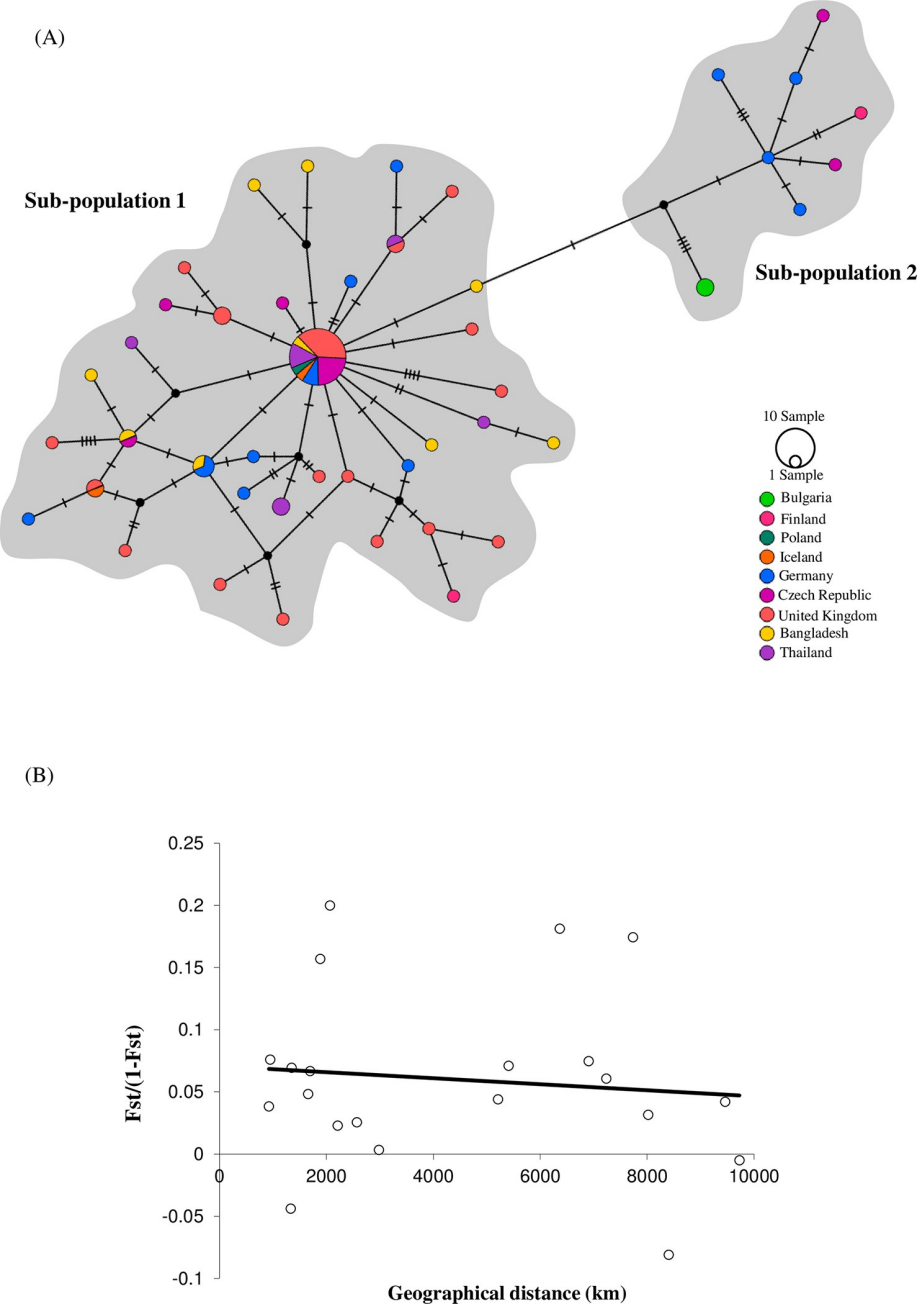
Divergence of populations of *Echinostoma revolutum* (s.s) across Eurasia. (A) Haplotype network based on the most parsimonious geneology of *nad*1 sequences of *E*. *revolutum* (*s*.*s*.) from across Eurasia. Pie charts are scaled to represent the number of individual parasites sharing a particular *nad*1 haplotype (see scale for sizing); colours indicate geographical location (key); black circles represent hypothetical ancestral haplotypes. (B) Mantel test regression analysis showing a lack of significant correlation between genetic distance and geographical distance between *E*. *revolutum* (*s*.*s*.) isolates across Eurasia.

Haplotype network analyses (Fig 3A) demonstrated lack of distinct geographically specific lineages between haplotypes of *E*. *revolutum* (*s*.*s*.). Many haplotypes were shared between European countries and between Europe and Asia (sub-population 1). The most common haplotype, representing 21/73 *E*. *revolutum* (*s*.*s*.) sequences, was shared between the UK, Czech Republic, Germany, Iceland, Poland, Thailand and Bangladesh. The haplotype network illustrated a low number of mutations between each haplotype with the majority of haplotypes separated by only 2–5 mutations, indicating high levels of gene flow and/or potential radiation. Furthermore, the haplotype network revealed some evidence of a potential second subpopulation made up of a cluster of unique haplotypes from Finland, Germany, Czech Republic and Bulgaria, separated from the main population by 8–11 mutations. The Mantel test for genetic isolation by distance revealed no significant correlation between genetic and geographical distance between *E*. *revolutum* (*s*.*s*.) isolates across Europe and Asia (Mantel test: y = -2E-06x + 0.0707, r^2^ = 0.0113; P = 0.722, P>0.05) (Fig 3B). There was no evidence of any major divergence between populations from across Eurasia and a lack of any isolation between populations.

### Evidence of historical demographic changes in *Echinostoma revolutum* (*s*.*s*.) populations

Tajima’s *D* values were negative for the Eurasia, Europe and Asia *nad*1 datasets, and were significant (*P*<0.001) for Eurasia and Europe (Tajima’s *D*: Eurasia *P* = 0.000001; Europe *P* = 0.000001) but not Asia (*P* = 0.039). Fu’s *Fs* was negative and significant across all datasets (Fu’s *Fs*: Eurasia *P* = 0.000001; Europe *P* = 0.000001; Asia *P* = 0.000001). These parameters indicate an excess of low frequency haplotypes compared to that expected under neutrality due to historical expansion in the *E*. *revolutum* (*s*.*s*.) population across Eurasia or selection processes (Table 3).

**Table 3:** Neutrality and mismatch tests across major population groupings of *Echinostoma revolutum* (*s*.*s*.) from Eurasia.

Group	No. sequences	*h*	*Hd*	Tajima’s *D*	Fu’s *Fs*	Raggedness (*r*)
**Eurasia**	73	45	0.917 (SD±0.029)	-2.32752*	-51.317 *	0.0088!
**Europe**	56	36	0.909 (SD±0.035)	-2.18457*	-33.210*	0.0102!
**Asia**	17	14	0.956 (SD±0.044)	-1.7009	-10.460*	0.0593

* *P*≤0.00;! P≤0.01 for *r*; *h*: haplotype numbers; *Hd*: haplotype diversity

**Fig 4:**
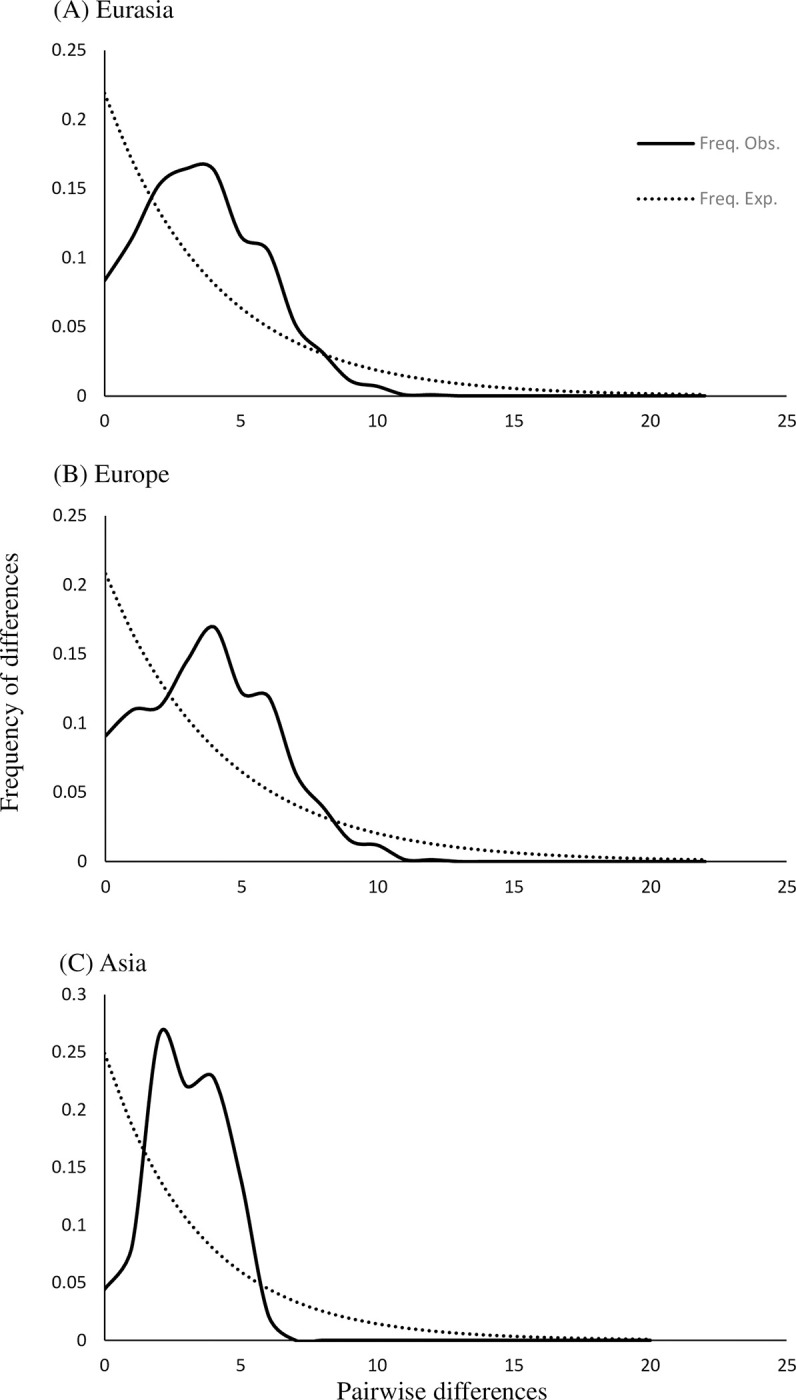
Mismatch distribution curves for *nad*1 haplotypes of *Echinostoma revolutum* (*s*.*s*.) isolates across Eurasia. **(**A) Eurasia, combined data from Europe and Asia. (B) Isolates from Europe including those from England. (C) Asian isolates from Bangladesh and Thailand.

The pairwise mismatch distributions for *E*. *revolutum* (*s*.*s*.) populations in Eurasia and Europe, however, appeared bimodal (Fig 4A and 4B). The raggedness index *r* suggested that the observed data differed significantly from expected values (Eurasia *P* = 0.002; Europe *P* = 0.005, both *P*<0.01) and therefore the Eurasian and European populations represent stable populations that have not undergone any major population expansion events; the pattern seen could be caused by selection and/or sub-population structuring. The Asia dataset was characterised by a high frequency of haplotypes and a non-significant raggedness index, suggesting a unimodal distribution (Asia *P* = 0.226, *P*>0.01) and supporting a hypothesis of population expansion having recently occurred in Asia.

### Detection of signatures of selection in *nad*1 sequences of *Echinostoma revolutum* (*s*.*s*.)

**Fig 5:**
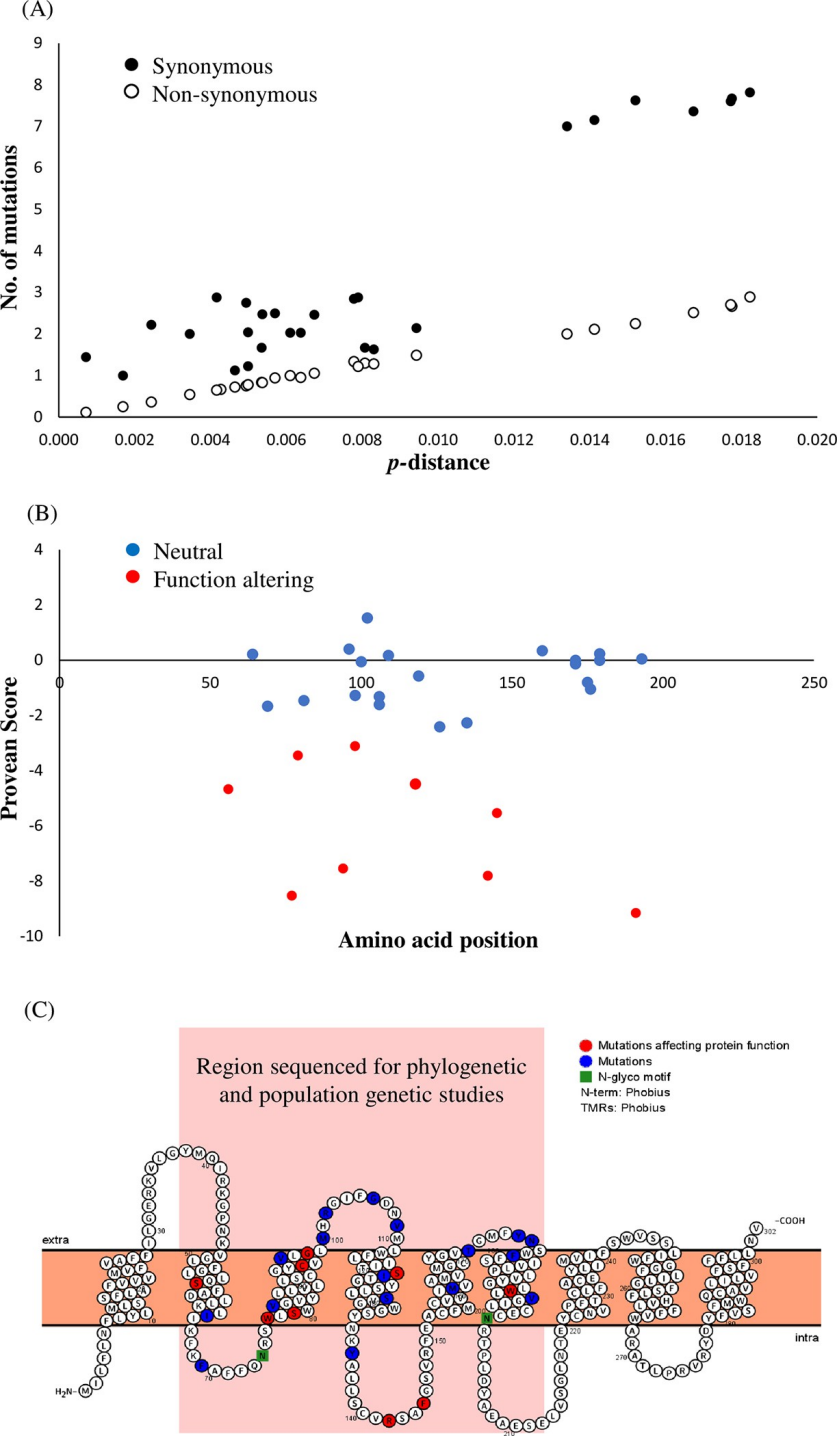
Detection of selection and the impact of amino acid replacements on *Echinostoma revolutum* (*s*.*s*.) *nad*1. **(**A) Contribution of synonymous and non-synonymous mutations to divergence (*p*-distance) between sequences within the Eurasian alignment based on pairwise differences between individual sequences within an alignment. (B) Provean analyses showing the distribution of amino acid replacements with all those with a score ≥-2.5 indicating a potential function altering change. (C) Protter 2D model of the *nad*1 protein illustrating the position and domains within which amnio acid replacements are occurring.

Initial analyses of standard dN/dS ratios across the Eurasian, European and Asian *E*. *revolutum* (*s*.*s*.) alignments showed that all geographical isolates appeared to be under purifying selection with dN/dS<1 (Eurasia dN/dS = 0.144; Europe dN/dS = 0.129; Asia dN/dS = 0.19). Owing to low sensitivity of simple alignment based dN/dS ratios, analyses per sequence were performed to identify if individual sequences differed from each other in terms of their content of synonymous and non-synonymous mutations across the Eurasian dataset. Synonymous mutations appeared to have the greatest contribution to *p*-distance across the data sets with substantially more synonymous mutations than non-synonymous mutations at higher *p*-distance values (Fig 5A). The codon-based Z test for positive selection identified only five sequence comparisons to be significantly different at *P*<0.05 allowing the rejection of the null hypothesis of strict neutrality (dN = dS)i (dN>dS) (S5 Table). Significant Z scores were identified between comparison of sequences from Germany representing haplotypes within sub-population 2 and haplotypes from Bangladesh and the UK (Fig 3A). Provean analyses of unique protein sequences from across Eurasia identified nine amino acid replacements with potential function altering properties at amino acid positions 56, 77, 79, 94, 98, 118, 142, 145 and 191 (Fig 5B). Protter analyses showed that the majority of the amino acid mutations were in transmembrane regions (Fig 5C). The two mutations at 142 and 145, however, were within a domain directly interacting with the interior of the mitochondria. Combined results indicate that there is some evidence of selection taking place within the Eurasian *E*. *revolutum* (*s*.*s*.) sequence data set.

## Discussion

Knowledge of the factors that affect the genetic structure within and among populations of zoonotic parasites and their phylogenetics is important for understanding their ecological and evolutionary processes and has consequences for monitoring of disease and parasite control [45]. Here, we used phylogenetic analysis to reveal the occurrence of *E*. *revolutum* (*s*.*s*.) in England, in addition to other echinostomatid species *Ep*. *aconiatum*, *Ep*. *recurvatum* and *H*. *conoideum*. Snail host ranges concur with data from previous molecular sampling programmes in Europe [11, 14, 15, 19, 27, 46], although *A*. *balthica* and *R*. *auricularia* are reported as new hosts for *Ep*. *aconiatum* and *H*. *conoideum*, respectively. Previous molecular studies on cercariae of trematodes in the UK have reported *E*. *recurvatum* from *A*. *balthica*, *Isthmiophora melis* [11] and *Echinostoma* sp. IG from *Planorbis* sp. in Wales [14]. The echinostomatid species belong to the transmission guild of waterfowl parasites that use Anatidae as definitive hosts [46], which were evident at all infected sites. *Echinostoma revolutum*, *Ep*. *recurvatum* and *H*. *conoideum* are agents of human echinostomiasis [3], but no autochthonous cases have been recorded yet in the UK.

Application of the K/θ species criterion supports previous studies using *nad*1 barcoding that there is lack of gene flow between Eurasian *E*. *revolutum* (*s*.*s*.) and USA *E*. *revolutum* and they are, therefore, genetically separate lineages [17, 18]. The cercariae of the two species differ morphologically and further work is required to describe USA *E*. *revolutum* as a new species when adults are available for examination [19]. We have provided new evidence that *E*. *revolutum* (*s*.*s*.) has a panmictic population in which genetic mixing occurs across Eurasia, probably due to movement of migratory avian hosts. There was low diversity within and between *E*. *revolutum* (*s*.*s*.) populations from Europe and Asia and no significant relationship between genetic divergence and geographical distance between isolates as revealed by the Mantel test. Furthermore, the haplotype network analyses illustrated that there were no geographically distinct genetic lineages of *E*. *revolutum* (*s*.*s*.) with a substantial number of haplotypes being shared not only across Europe, but between European and Asian populations of *E*. *revolutum* (*s*.*s*.), indicating gene flow. This lack of differentiation between *E*. *revolutum* (*s*.*s*.) populations most likely reflects the flyways of aquatic avian hosts. Many species of ducks, geese and swans overwinter in Southeast Asia and then migrate to breed in Northern Eurasia, mixing with birds from Europe [47]. The importance of avian host movement and connectivity in the distribution and homogenisation of pathogen genotypes is documented; for example, there is a direct link between bird intercontinental migration and the distribution of avian influenza viruses [48, 49]. Trans-hemispheric gene flow of the avian schistosome *Trichobilharzia querquedulae* has been demonstrated by lack of phylogenetic structuring between populations [50].

Interestingly, when *E*. *revolutum* (*s*.*s*.) sequence data sets for Europe and Asia were analysed independently from each other, the European dataset showed the same patterns as the combined Eurasian sequences. Significant negative values for Tajima’s *D* and Fu’s *Fs* suggested demographic population expansion, but mismatch distribution analyses supported a stable population. The conflict between the Tajima’s *D*, Fu’s *Fs* and raggedness scores can occur as a result of sub-population structuring and/or the presence of selection. The haplotype network analyses indicated the presence of a potential sub-population composed of haplotypes specific to central and northern Europe. Similarly, the codon-based Z test for the detection of selection indicated that the majority of significant differences in dN/dS occurred through comparisons of haplotypes within sub-population 2. This sub-population may be the result of local adaptations in a population of *E*. *revolutum* (*s*.*s*.) which is resident to central and northern Europe. Although the Eurasian population is panmictic, it could be argued that even with gene flow between countries across Asia and Europe, sub-populations could have emerged as the parasites establish in non-migratory bird and mammal populations. This pattern of local adaptation and emergence of sub-populations has also been observed in other parasites which use migratory animals as hosts. The same trend was seen in populations of *Schistosoma turkestanicum* with evidence of continued migration and gene flow from Asia, but also local adaptation showing periods of isolation and establishment in local host populations [51].

Significant negative values for Fu’s *Fs* and a unimodal mismatch distribution analysis supported recent expansion of the Asia population. There is no direct evidence to explain this possible demographic change, but there may be a connection between the importance of free grazing ducks as reservoir hosts for echinostomes, including *E*. *revolutum* [52], and intensification of duck production in Asia [53]. In Thailand, for example, most farmed ducks are raised in intensive free-grazing production systems where they are rotated between post-harvest rice paddy fields also used by wild birds [52, 54]. The fields, therefore, act as potential spillover hotspots. However, to resolve the evolutionary history and population structure of *E*. *revolutum* (*s*.*s*.), further intensive sampling of the parasites from an increased number of countries across Eurasia is required with deeper and more extensive genome sequencing, considering genetic signals from multiple genes in the mitochondrial genome and across the nuclear genomes of these parasites [28].

The codon-based Z test for positive selection identified the presence of some positive selection associated with comparisons between individual haplotypes between sub-populations 1 and 2 and between haplotypes from UK and Bangladesh. Provean and Protter analyses revealed possible function-altering mutations in the *nad*1 protein within domains that are exposed to the interior of the mitochondria, indicating the presence of a potential adaptive response. The *nad*1 is part of the oxidative phosphorylation (OXPHOS) system complex1 of the mitochondria and is the first protein in the electron transport chain. Studies have illustrated adaptation in this gene as an adaptive response in organisms to either cold temperatures, seasonal change or living at different altitudes [55–60]. Adaptations within mitochondrial genes in trematodes have only been illustrated in *S*. *turkestanicum* [51] and within the *Schistosoma indicum* group [60]. Both studies showed that functional changes within key domains of mitochondrial genes were seen in different isolates and populations of schistosomes either living at higher altitudes or in both tropical and temperate environments. It was suggested that the response in mitochondrial genes was an adaptive response to low temperatures, allowing electron leakage and potentially enabling parasites to withstand colder water environments [51, 60]. This may account for the pattern seen in the *E*. *revolutum* (*s*.*s*.) dataset as central and northern Europe are known to experience cold winters, thus any established populations within these areas would have adapted to local conditions, ultimately giving rise to sub-population 2. Similarly, this may also account for some of the differences seen between the sequences of *E*. *revolutum* (*s*.*s*.) from the UK and Bangladesh, with some local adaptations to the cooler temperate environments of the UK versus the subtropical monsoon climate of Bangladesh. Although *E*. *revolutum* (*s*.*s*.) may represent a large interconnected panmictic population in Eurasia, there is some local adaptation occurring potentially as a result of balancing selection despite constant gene flow.

## Conclusions

The present study provides new molecular evidence for a panmictic population of the zoonotic parasite *E*. *revolutum* (*s*.*s*.) in Eurasia in which genetic flow is probably due to movements of migratory waterfowl. We present evidence for likely recent demographic expansion of *E*. *revolutum* (*s*.*s*.) in Asia, although the Eurasian population appears to be stable with evidence of sub-population structure and some selection. In addition, we provide novel molecular data on species richness of echinostomatids and their snail hosts in England and phylogeographically expand the *nad*1 database for identification of echinostomatids. Collectively, the data offer important insights into the molecular epidemiology of *E*. *revolutum* (*s*.*s*.) in the context of disease dynamics and contribute a valuable framework for further studies into the parasite and factors affecting its distribution.

## Supporting information

S1 Fig Phylogenetic tree from maximum likelihood analysis based on *cox*1 sequences of snail hosts. Shaded clades indicate inclusion of newly generated sequences. The scale shows the number of nucleotide substitutions per site between DNA sequences. Species clades shown as collapsed.
(TIF)

S1 Table Summary data for echinostomatid and snail host isolates used for generation of novel *nad*1 and *cox*1 sequences. (XLSX)

S2 Table Summary data for *cox*1 sequences of snails downloaded from GenBank and used in molecular analyses. (XLSX)

S3 Table Summary data for *nad*1 sequences of echinostomatids downloaded from GenBank and used in molecular analyses. (XLSX)

S4 Table Summary data for *Echinostoma revolutum* (*s*.*s*.) isolates from Europe and Asia used in molecular diversity and population structure analyses. (XLSX)

S5 Table Z scores for codon selection in *Echinostoma revolutum* (*s*.*s*.) analyses. (XLSX)

S6 Table Host and morphological characteristics of *Echinostoma revolutum* (*s*.*s*.), *Echinoparyphium recurvatum* and *Echinoparyphium aconiatum* cercariae in the present study. (DOCX)
