# Supplementary figures and images for: Molecular epidemiological analyses reveal extensive connectivity between Echinostoma revolutum (sensu stricto) populations across Eurasia and species richness of zoonotic echinostomatids in England
Source: PLoS One. 2023 Feb 6;18(2):e0270672. doi: 10.1371/journal.pone.0270672 (PMC9901765; doi:10.1371/journal.pone.0270672)

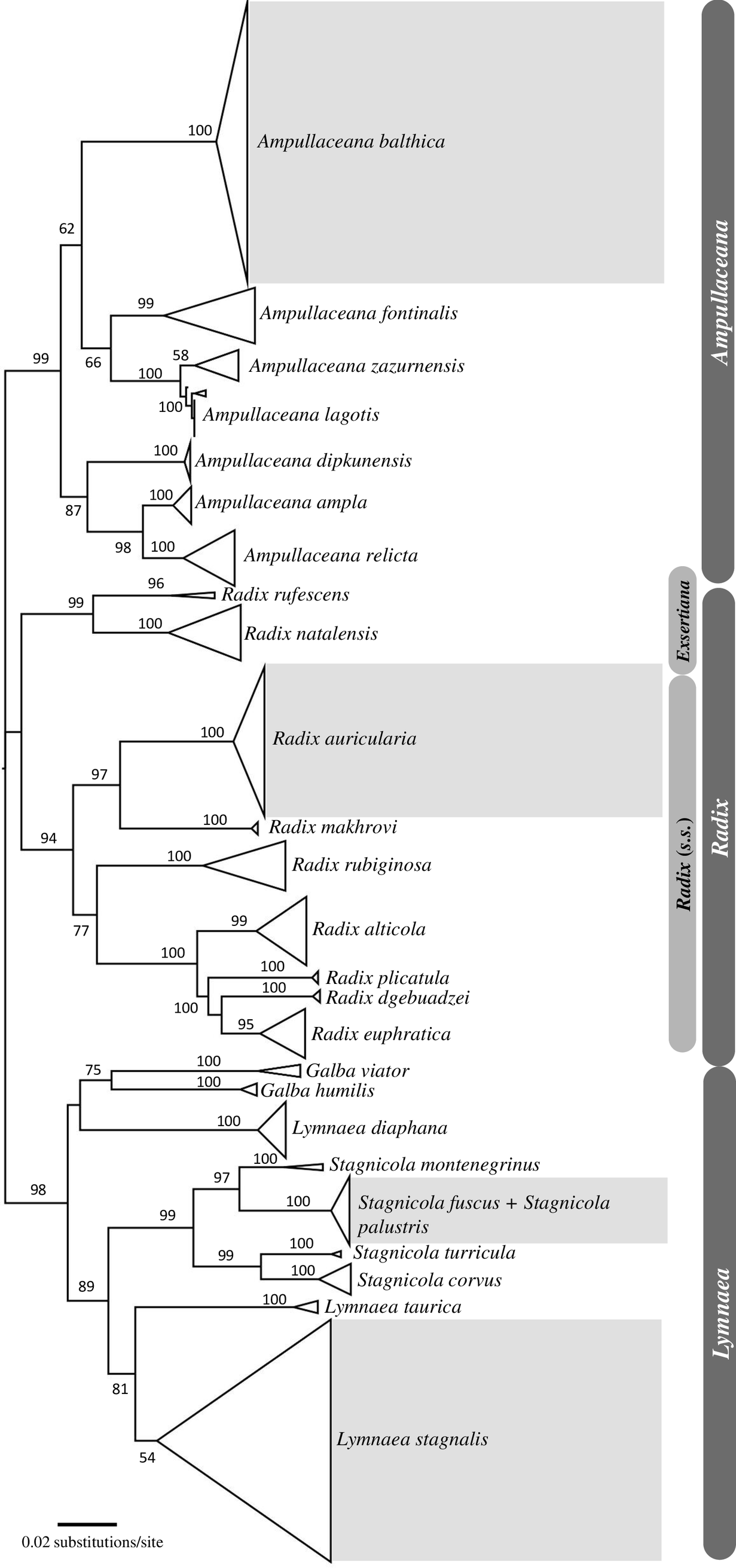

Supplement: S1 Fig — Shaded clades indicate inclusion of newly generated sequences. The scale shows the number of nucleotide substitutions per site between DNA sequences. Species clades shown as collapsed. (TIF) [file pone.0270672.s001.tif]
