# Supplementary material for: Molecular epidemiological analyses reveal extensive connectivity between Echinostoma revolutum (sensu stricto) populations across Eurasia and species richness of zoonotic echinostomatids in England
Source: PLoS One. 2023 Feb 6;18(2):e0270672. doi: 10.1371/journal.pone.0270672 (PMC9901765; doi:10.1371/journal.pone.0270672)
Supplement: S6 Table — (DOCX) [file pone.0270672.s007.docx]

**S6 Table. Host and morphological characteristics of *Echinostoma revolutum* (*s.s.*), *Echinoparyphium recurvatum* and *Echinoparyphium aconiatum* cercariae in the present study.** Measurements in μm are given as a range followed by the mean in parentheses based on specimens fixed in 4% formalin* or live specimens**.

***Echinostoma revolutum* (Frölich, 1802) Rudolphi, 1809 (*sensu stricto*)**

First intermediate hosts: *Ampullaceana balthica* (Linnaeus, 1758), *Lymnaea stagnalis* (Linnaeus, 1758), *Radix auricularia* (Linnaeus, 1758), *Stagnicola palustris* (O.F. Müller, 1774) (this study). Characteristic features: 37 collar spines, 12 paraoesophageal glands stain with neutral red, conspicuous cystogenous cells post-pharynx to posterior extremity of body, simple tail with contractile narrow tip and seven small tegumental finfolds.

Registration numbers: NHM UK 2018.11.14.2; NHM UK 2021.11.16.9

GenBank accession numbers: ON653218-ON653243

| **Morphological features** | **This study**  **n = 20*** | **Georgieva *et al* 2013 n=11*** | **Faltýnková *et al* 2015 *** |
| --- | --- | --- | --- |
| Body length | 149-231 (178) | 159-234 (188) | 177-220 (194) |
| Body width (maximum) | 111-135 (122) | 107-125 (112) | 106-128 (119) |
| Tail length | 345-376 (360) | 316-405 (367) | 343-416 (396) |
| Tail width at base | 32-44 (40) | 20-36 (27) | 34-41 (38) |
| Subterminal oral sucker  length x width | 31-43 (40) x 35-49 (42) | 38-52 (45) x 37-49 (42) | 38-48 (42) x 38-48 (42) |
| Collar width | 75-93 (85) | - | 66-83 (77) |
| Post-equatorial ventral sucker  length x width | 34-50 (48) x 43-49 (42) | 47-66 (55) x 48-60 (54) | 43-58 (50) x 47-56 (51) |
| Oesophageal primordium length | 32-45 (38) | 30-55 (40) | 31-45 (38) |

Faltýnková A, Georgieva S, Soldánová M, Kostadinova A. A re-assessment of species diversity within the ‘revolutum’ group of *Echinostoma* Rudolphi, 1809 (Digenea: Echinostomatidae) in Europe. Syst Parasitol. 2015; 1: 1–25. doi: 10.1007/s11230-014-9530-3.

Georgieva S, Selbach C, Faltýnková A, Soldánová M, Sures B, Skírnisson K, Kostadinova A. New cryptic species of the 'revolutum' group of Echinostoma (Digenea: Echinostomatidae) revealed by molecular and morphological data. Parasit Vectors. 2013,6: 64. doi: 10.1186/1756-3305-6-64.

***Echinoparyphium recurvatum* (Linstow, 1873) Dietz, 1909**

First intermediate hosts: *Ampullaceana balthica* (Linnaeus, 1758) (Ab), *Radix auricularia* (Linnaeus, 1758) (Ra) (this study). Characteristic features: 45 collar spines, no paraoesophageal glands, ventral depression surrounding ventral sucker, conspicuous cystogenous cells post-pharynx to posterior extremity of body, simple tail with blunt tip and without tegumental finfolds.

Registration numbers: NHM UK 2018.11.14.4; NHM UK 2021.11.16.11

GenBank accession numbers: ON653266-ON653296

| **Morphological features** | **This study**  **n = 20*** | **Pantoja *et al* (2021) n = 25*** | **Pantoja *et al* (2021)**  **n = 6**** |
| --- | --- | --- | --- |
| Body length | 268-349 (313) | 219-319 (275) | 327-376 (351) |
| Body width (maximum) | 110-131 (121) | 123-196 (147) | 168-201 (185) |
| Tail length | 526-640 (587) | 269-451 (383) | 399-389 (447) |
| Tail width at base | 40-46 (45) | 36-53 (44) | 45-67 (55) |
| Subterminal oral sucker  length x width | 27-41 (35) x 32-41 (36) | 35-52 (44) x 39-54 (47) | 41-56 (49) x 48-64 (55) |
| Collar width | 81-90 (86) | 76-118 (90) | 110-141 (126) |
| Post-equatorial ventral sucker  length x width | 34-48 (40) x 37-52 (43) | 41-67 (54) x 48-82 (62) | 53-70 (61) x 53-82 (68) |
| Oesophageal primordium length | 82-118 (116) | - | - |

Pantoja C, Faltýnková A, O’Dwyer K, Jouet D, Skírnisson, Kudlai O. Diversity of echinostomes (Digenea: Echinostomatidae) in their snail hosts at high latitudes. Parasite. 2021; 28: 59. doi: org/10.1051/parasite/2021054.

***Echinoparyphium aconiatum*** **Dietz, 1909**

First intermediate hosts: *Ampullaceana balthica* (Linnaeus, 1758), *Lymnaea stagnalis* (Linnaeus, 1758), *Radix auricularia* (Linnaeus, 1758) this study). A detailed morphological description linked to DNA sequences is not available for *Ep. aconiatum* and therefore morphometric comparisons are not valid.

Characteristic features: 37 collar spines, no paraoesophageal glands, ventral depression surrounding ventral sucker, conspicuous cystogenous cells post-pharynx to posterior extremity of body, simple tail with sub-terminal groove-like depression and without tegumental finfolds.

Registration numbers: NHM UK 2018.11.14.3; NHM UK 2021.11.16.10

GenBank accession numbers: ON653244-ON653265

| **Morphological features** | **This study**  **n = 20*** |
| --- | --- |
| Body length | 310-484 (404) |
| Body width (maximum) | 165-218 (184) |
| Tail length | 623-946 (799) |
| Tail width at base | 30-46 (38) |
| Subterminal oral sucker  length x width | 20-29 (25) x 21-25 (30) |
| Collar width | 92-114 (102) |
| Post-equatorial ventral sucker  length x width | 30-53 (41) x 39-68 (52) |
| Oesophageal primordium length | 148-233 (179) |
